# Quantitative ultrasound assessment of the effect of parity on bone mineral density in females

**DOI:** 10.1186/s12905-021-01516-w

**Published:** 2021-10-30

**Authors:** Shahnaz Akil, Huda Al-Mohammed, Norah Al-Batati, Maissa Tirsen, Ahad Al-Otaibi, Aram AlZahrani, Deena Bakhder, Ruba AlSubaie, Samar AbuAlsaud

**Affiliations:** 1 Department of Radiological Sciences, College of Health and Rehabilitation Sciences, Princess Nourah Bint Abdulrahman University, Riyadh, Saudi Arabia; 2 Department of Laboratory Medicine, Clinical Physiology, Karolinska Institutet, Huddinge, Sweden; 3 Department of Clinical Physiology, Karolinska University Hospital, 141 86 Stockholm, Sweden

**Keywords:** Parity, Ultrasound, Females, Osteoporosis, Bone mineral density, Pregnancy, Breastfeeding

## Abstract

**Background:**

The effect of pregnancy and breastfeeding on a female’s bone mineral density (BMD) is controversial. This prospective study aims to investigate the effect of parity on BMD among pre-menopausal multiparous females using quantitative ultrasound as a screening method and females with no pregnancies (nulliparous) as a control group.

**Methods:**

A portable ultrasound-based bone densitometer (DMS PEGASUS SMART, Mauguio, France) was used to indirectly assess the BMD in 51 multiparous (29–45 years) and 51 nulliparous Arabic females (18–35 years) by quantifying the broadband ultrasound attenuation (BUA) from their right calcaneus bone. BUA > 70 db/mhz = normal, BUA 65–69.9 db/mhz = below average, BUA 55–64.9 db/mhz = osteopenia and BUA < 55 db/mhz = osteoporosis.

**Results:**

There was a significant difference in mean BUA between multiparous and nulliparous females (74.1 db/mhz vs. 69.3 db/mhz, p = 0.006). The prevalence of normal BMD was significantly higher in the nulliparous group than in the multiparous group (70.6% vs. 47.1%, p = 0.02). Osteoporosis was found in the multiparous group only (3/51). Among the multiparous females who breastfed (43/51), a total of 51.2% (22/43) had normal BMD, 25.6% (11/43) had BMD below average, 18.6% (8/43) had osteopenia and 4.7% (2/43) had osteoporosis. No significant differences in mean BUA (p = 0.2) were found between the group of females who breastfed for one year (13/43; BUA: 70.5 ± 9.4), the group of females who breastfed for 6–11 months (8/43; BUA: 70.6 ± 10.0) and those who breastfed for less than six months (22/43; BUA: 71.6 ± 9.4). A binary logistic regression model built for predicting BMD normality showed significance for the variable parity (p = 0.03), while the effect of the possible confounding variables BMI and age on BMD normality was found to be non- significant (p = 0.1 and p = 0.6, respectively).

**Conclusion:**

Parity affects the BMD, as assessed by a portable ultrasound-based bone densitometer, of young and middle-aged females as compared to the BMD of nulliparous females.

**Supplementary Information:**

The online version contains supplementary material available at 10.1186/s12905-021-01516-w.

## Background

Pregnancy, birth and lactation cause several changes in bone metabolism, which might have both short- and long-term effects on a female’s health. The increased calcium mobilization and bone reabsorption that occur during pregnancy and lactation can result in calcium deficiency [1]. Calcium deficiency in the body can cause a prolonged imbalance of calcium in the bone and may induce either osteopenia or osteoporosis if the deficiency is not treated [1].

Osteoporosis is a skeletal disorder that has burdened the global economy due to its high incidence especially in the female population [2]. The disorder is characterized by a reduction in bone strength resulting in increased fracture risk due to a decrease in bone mineral density (BMD) [3]. Therefore, an abnormal BMD may indicate the presence of osteoporosis [3, 4]. Several diagnostic methods exist for the assessment of BMD with dual-energy X-ray absorptiometry (DXA) being the most widely used method [5, 6]. Another non-invasive method that can be used in the assessment of BMD is an ultrasound-based bone densitometer named quantitative ultrasound (QUS) [7–9]. Compared to DXA, QUS is radiation-free, cheaper and widely available [8]. Using QUS, an indirect assessment of BMD is possible by quantifying the broadband ultrasound attenuation (BUA in db/mhz) or an ultrasound index [8]. Therefore, QUS may be used as a screening method to assess the risk of osteoporosis.

Several previous studies have found no association between the number of pregnancies (parity) and increased risk of osteoporosis, despite the concerns regarding the effect of the number of pregnancies on the BMD of females [1, 10–14]. Some previous studies have even shown an improvement in the BMD of females who have given birth to more than one child (multiparous) [15–17]. However, studies that examine the long-term effect of parity on BMD, as assessed by a portable ultrasound machine, in pre-menopausal females compared to a control group of nulliparous females in a Saudi population are lacking. Therefore, this prospective study aims to investigate the effect of parity on BMD among pre-menopausal multiparous females using quantitative ultrasound as a screening method as well as females with no pregnancies as a control group.

## Methods

### Study population and design

A prospective and observational study was performed at Princess Nourah Bint Abdulrahman University in Riyadh, Saudi Arabia from January 2019 to March 2019. A total of 102 pre-menopausal Arabic females who were either staff or students at Princess Nourah Bint Abdulrahman University were included. Of these, a total of 51 had given birth to more than one child (multiparous group) and 51 did not have children (nulliparous group). The following inclusion criteria were used: female gender, general good health, age between 18 and 45 years old, no children for the control group and 2–3 children for the multiparous group. To exclude any possible variables that may affect BMD and hence act as confounding factors, the following exclusion criteria were applied: post-menopausal females, females on calcium supplementation, volunteers who according to their known medical history had diabetes, hyperthyroidism, asthma, vitamin D deficiency as well as females who were pregnant or breastfeeding at the time of the study.

### Quantitative ultrasound and BMD normality

A portable ultrasound-based bone densitometer (DMS PEGASUS SMART bone densitometer, Mauguio, France) [18] was used for the indirect assessment of BMD. Using the ultrasound- based bone densitometer, a parameter named broadband ultrasound attenuation (BUA) was quantified from the right calcaneus bone of all volunteers. All 102 ultrasound studies were performed using the same ultrasound machine. The volunteers in the multiparous and control group were then classified into one of the following four BMD normality categories based on their recorded BUA, as previously suggested [19] and as recommended by the manufacturer guidelines [18]: Normal (BUA > 70 db/mhz), below average (BUA 65–69.9 db/mhz), osteopenia (BUA 55–64.9 db/mhz) and osteoporosis (BUA < 55 db/mhz). A questionnaire, that was developed for this study (see Additional file 1), was used to collect baseline data including the number of children, weight, length, age, history of breastfeeding and family history of osteoporosis.

### Statistical analysis

The software Graph Pad Prism version 8.0 (Graph Pad Software, Inc., La Jolla, CA, USA) was used for all statistical analyses. Student’s t-test was used to assess if there is a significant difference in mean BUA between the control group and the multiparous group. A One-Way Analysis of variance (ANOVA) was used to test if there were significant differences in mean BUA between females who breastfed for one year, those who breastfed for 6–11 months and those who breastfed for less than six months. Furthermore, a CHI-2 test was used to assess the difference between the multiparous and control group in the number of females in each of the BMD normality categories.

A binary logistic regression model was used to assess if other variables than parity affected the BMD normality of the studied population. In the model, the four BMD normality categories were recoded into two categories: Normal and abnormal (below average, osteoporosis, osteopenia). The potential predictors which were included in the model were parity (nulliparous/multiparous), body mass index (BMI) and age. A p-value ≤ 0.05 was considered statistically significant.

## Results

For the multiparous group, the timings of QUS in relation to dates of pregnancy and lactation were 6.4 ± 4.3 years and 7.3 ± 4.4 years, respectively. Baseline characteristics for the included volunteers can be seen in Table 1. None of the included females had reported that they were on calcium supplementation.

**Table 1:** Population characteristics

	Multiparous	Nulliparous	Total population
Number of volunteers	51	51	102
Age (years)	35 ± 4; range: 29–45; median: 35; Q1: 33; Q3: 40	30 ± 4; range 18–39; median: 21; median: 21; Q1: 21; Q3: 23	32 ± 4; range 18–45
Height (cm)	159 ± 6	160 ± 7	160 ± 7
Weight (kg)	72 ± 12	61 ± 15	66 ± 14
BMI (kg/m^2^)	29 ± 5	24 ± 5	27 ± 5
Number of children			
2 children	45% (23/51)	0%	23% (23/102)
3 children	55% (28/51)	0%	27% (28/102)
Breastfed their children	84% (43/51)	0%	42% (43/102)
Family history of osteoporosis	22% (11/51)	12% (6/51)	17% (17/102)

Data is expressed as mean ± SD

### Effect of parity on BUA and BMD normality

**Fig. 1:**
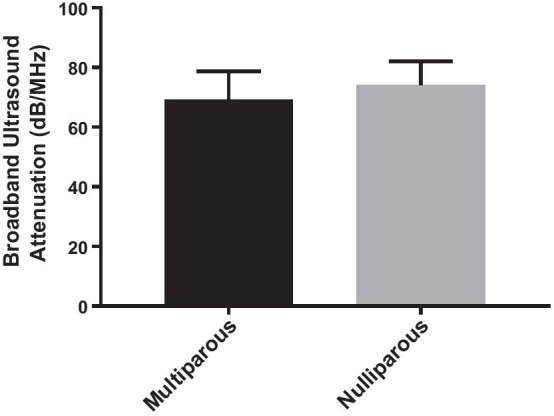
Comparison of the mean broadband ultrasound attenuation (BUA) value, as assessed by an ultrasound-based bone densitometer, of females who had no pregnancies (nulliparous) with the mean BUA of females in the multiparous group. The mean BUA is higher in the nulliparous group reflecting a higher bone mineral density in this group as compared to the multiparous group

The BUA values ranged between 60 and 102 db/mhz in the nulliparous group and between 37.4 and 85.1 db/mhz in the multiparous group. There was a significant difference in mean BUA between females who had no pregnancies (nulliparous) and females in the multiparous group (74.1 db/mhz vs. 69.3 db/mhz, p = 0.006), as can be seen in Fig. 1.

**Fig. 2:**
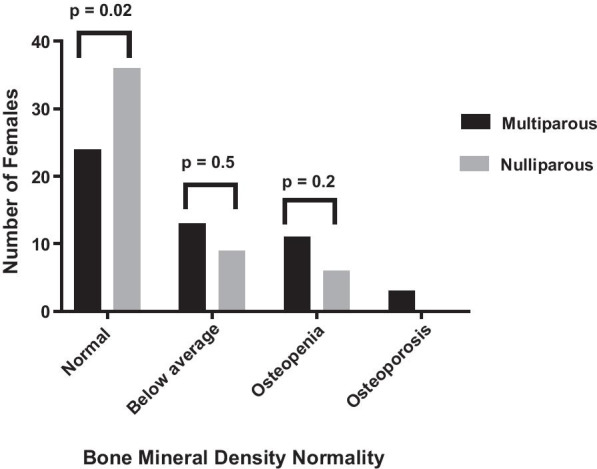
Comparison of the number of females in the multiparous and nulliparous group in each of the bone mineral density normality categories (normal, below average, osteopenia and osteoporosis). A p-value ≤ 0.05 was considered statistically significant

The number of females in each of the three BMD normality categories for the nulliparous and multiparous group can be seen in Fig. 2. The prevalence of normal BMD was significantly higher in the nulliparous group than in the multiparous group (70.6% vs 47.1%, p = 0.02), as can be seen in Fig. 2. No significant difference was found between the nulliparous and the multiparous group in the prevalence of a BMD below average (17.6% vs 23.5%, p = 0.5) and the prevalence of osteopenia (11.8% vs 21.6%, p = 0.2). In the nulliparous group, there were no cases of osteoporosis, while 6% (3/51) of the females in the multiparous group had osteoporosis. The binary logistic regression model built for predicting BMD normality showed significance for the variable parity (p = 0.03), while the effect of the possible confounding variables BMI and age on BMD normality was found to be non- significant (p = 0.1 and p = 0.6, respectively), as shown in Table 2.

**Table 2:** A binary logistic regression investigation of the relationship between different independent variables (age, BMI, parity) and the dependent variable bone mineral density normality that is converted to a binary scale (normal, abnormal)

	Dependent variable: bone mineral density normality (0 = below average, osteoporosis or osteopenia, 1 = normal)	
	Odds ratio (confidence interval)	p-value
Age (years)	1.022 (0.932–1.121)	0.6
BMI (kg/m^2^)	1.078 (0.989–1.175)	0.1
Parity (0 = nulliparous, 1 = multiparous)	0.187 (0.043–0.824)	0.03*

^*^Significant (p ≤ 0.05)

### Breastfeeding

The proportion of multiparous females who breastfed was 84.3% (43/51). Of these, 30% (13/43) continued to breastfeed for a full year, 18.6% (8/43) for 6–11 months and 51.1% (22/43) breastfed for less than six months. No significant differences in mean BUA (p=0.2) were found between the group of females who breastfed for one year (BUA: 70.5±9.4), the group of females who breastfed for 6–11 months (BUA: 70.6±10.0) and those who breastfed for less than six months (BUA: 71.6±9.4). A total of 51.2% (22/43) of the females who breastfed had normal BMD, 25.6% (11/43) had BMD below average, 18.6% (8/43) had osteopenia and 4.7% (2/43) had osteoporosis. The mean time that had passed after stopping the breastfeeding was 3.3 years ±2.1 (median: 3.5 years, Q1: 1 year, Q3: 5 years)

## Discussion

To the best of our knowledge, this is the first study to investigate the effect of parity on the BMD of young and middle-aged Arabic females using the portable ultrasound-based bone densitometer. Evidence from this study, showing that BMD may be affected by parity, encourages both enhanced education about bone health in multiparous females and further studies related to the assessment of fracture risk in this group of females.

### Effect of parity

In the present study, the significant difference in mean BUA values found between multiparous and nulliparous females (p = 0.006, Fig. 1) shows that females with no pregnancies had a better BMD than females with 2–3 pregnancies (multiparous). The majority of females with normal BUA values were within the nulliparous group (n = 36). In addition, no cases of osteoporosis were found in the nulliparous group, which further shows that the BMD of multiparous females is more affected. Therefore, the findings of the current study contradict findings from previous studies showing that multi-parity does not affect BMD [1, 13, 20, 21]. The results of this study may be explained by the high calcium demand during pregnancy, which could theoretically result in a long-term decrease in bone mass [16]. Other previous studies that have shown a positive or unchanged effect of parity on BMD [1, 11, 13, 20–22] suggest that the higher estrogen levels in the third trimester of pregnancy cause an increased absorption of calcium [23]. Some of the earlier studies did not observe any effect of parity on BMD [1, 21] as these studies included relatively older females with higher parity as compared to the current study. This study investigated the long-term effect of parity on BMD whereas previous studies that reported a positive or unchanged effect of parity on BMD were based on the short-term effect on BMD[1, 11, 13, 20–22]. In addition, in contrast to the current study, previous studies have used other bone sites (femoral neck, spine and hip) than the calcaneus bone to assess BMD [1, 11, 13, 20–22], which may explain the difference in findings. Furthermore, the results of the current study showed that the difference in BMD normality between the nulliparous and multiparous groups found in this study cannot be explained by a difference in BMI between the two groups (binary logistic regression model: p = 0.1). Previous studies have suggested that a higher BMI during pregnancy is protective against a decrease in BMD [24, 25].

### Breastfeeding

In this study, the high number of multiparous females who breastfed (43/51, Table 1) suggests that breastfeeding may have contributed to the significant difference in BUA found between the multiparous and nulliparous females. It has previously been found that BMD is reduced by approximately 5% during pregnancy and lactation [21]. However, the present study also showed that 51.2% (22/43) of the female who breastfed had normal BMD and most females who did not breastfeed (8/51) were within the osteopenia category (Fig. 2). This is in line with previous studies showing no prolonged effect of lactation on BMD [26]. The non- significant difference in BUA (p = 0.2), found in the current study, between females who breastfed for different durations is in line with the findings of some previous studies[1, 21, 27] but contradicts another study [28]. It must be noted that the maximum breastfeeding duration found in the current study was one year.

### Quantitative ultrasound as a screening method

In contrast to previous studies, ultrasound and not DXA was used to assess BMD. A previous study has shown that quantitative ultrasound is a useful tool to detect osteoporosis [29]. Another study in Japan showed that the introduction of ultrasound was associated with a 30% increase in the diagnosis of osteoporosis [30]. Any osteoporosis case has to, however, be confirmed by DXA which remains today’s gold standard [31]. On the other hand, the portable ultrasound machine may act as a useful radiation-free screening tool for those with suspected abnormal BMD or osteoporosis.

### Limitations

The following limitations should be considered when interpreting the results of this study. The non-significant difference found in this study in the prevalence of a BMD below average and osteopenia, between the nulliparous and multiparous group, may be due to the low number of females in the sub-categories. Given that most multiparous females in the included population chose to breastfeed, it is difficult to assess the effect of breastfeeding on BMD separately.

## Conclusion

Parity affects the bone mineral density, as assessed by a portable ultrasound-based bone densitometer, of young and middle-aged females as compared to a control group of nulliparous females. Therefore, awareness should be increased among females about the importance of screening for abnormal BMD with the radiation-free portable ultrasound machine post-partum to enhance early treatment.

## Supplementary Information

**Additional file 1.** A questionnaire used to collect baseline data of the volunteers.

## Data Availability

The datasets used and/or analyzed during the current study are available from the corresponding author on reasonable request.

## Abbreviations

BMD: Bone mineral density
DXA: Dual-energy X-ray absorptiometry
QUS: Quantitative ultrasound
BUA: Broadband ultrasound attenuation
