# Supplementary material for: Quantitative ultrasound assessment of the effect of parity on bone mineral density in females
Source: BMC Womens Health. 2021 Oct 30;21:380. doi: 10.1186/s12905-021-01516-w (PMC8557593; doi:10.1186/s12905-021-01516-w)
Supplement: Supplementary file 1 — Additional file 1. A questionnaire used to collect baseline data of the volunteers. [file 12905_2021_1516_MOESM1_ESM.docx]

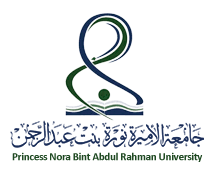


Ultrasound assessment of bone mineral density among multiparous and nulliparous females at Princess Nourah Bint Abdulrahman University

Q1: What is your age?

……….. years

Q2: What is your weight?

………… kg

Q3: What is your length?

………….. cm

Q4: Are you pregnant?

- Yes
- No

Q5: How many children do you have?

- 0
- 1
- 2-3
- More than 3

Q6: What are the ages of your children?

........... years

………… years

…………. years

Q7: Did you breastfeed all your children?

- Yes
- No

Q8: How many children have you breastfed if you did not breastfeed all your children?

………... children

Q9: How long have you breastfed your youngest child?

- 1 year
- 6 months
- Less than 6 months

Q10: Do you still have your period?

- Yes
- No

Q11: Do you follow a normal diet?

- Yes
- No

Q12. Are you healthy?

- Yes
- No

Q13: Indicate if you are diagnosed with any of the following conditions?

- Diabetes
- Hypertension
- Hyperthyroidism
- Lower estrogen level
- Asthma
- Vitamin D deficiency

Q14: Do you have family history of osteoporosis?

- Yes
- No
